# Coronavirus Infection and Cholesterol Metabolism

**DOI:** 10.3389/fimmu.2022.791267

**Published:** 2022-04-21

**Authors:** Jun Dai, Huan Wang, Ying Liao, Lei Tan, Yingjie Sun, Cuiping Song, Weiwei Liu, Xusheng Qiu, Chan Ding

**Affiliations:** ^1^ College of Animal Science and Technology, Guangxi University, Nanning, China; ^2^ Shanghai Veterinary Research Institute, Chinese Academy of Agricultural Sciences, Shanghai, China; ^3^ Experimental Animal Center, Zunyi Medical University, Zunyi City, China; ^4^ Jiangsu Co-Innovation Center for Prevention and Control of Important Animal Infectious Diseases and Zoonoses, Yangzhou University, Yangzhou, China

**Keywords:** coronavirus, cholesterol, metabolism dysregulation, immune response, therapy

## Abstract

Host cholesterol metabolism remodeling is significantly associated with the spread of human pathogenic coronaviruses, suggesting virus-host relationships could be affected by cholesterol-modifying drugs. Cholesterol has an important role in coronavirus entry, membrane fusion, and pathological syncytia formation, therefore cholesterol metabolic mechanisms may be promising drug targets for coronavirus infections. Moreover, cholesterol and its metabolizing enzymes or corresponding natural products exert antiviral effects which are closely associated with individual viral steps during coronavirus replication. Furthermore, the coronavirus disease 2019 (COVID-19) caused by severe acute respiratory syndrome coronavirus 2 infections are associated with clinically significant low cholesterol levels, suggesting cholesterol could function as a potential marker for monitoring viral infection status. Therefore, weaponizing cholesterol dysregulation against viral infection could be an effective antiviral strategy. In this review, we comprehensively review the literature to clarify how coronaviruses exploit host cholesterol metabolism to accommodate viral replication requirements and interfere with host immune responses. We also focus on targeting cholesterol homeostasis to interfere with critical steps during coronavirus infection.

## Introduction

Coronaviruses are enveloped viruses with non-segmented, single-stranded, positive sense RNA genomes (1). They belong to the *Nidovirales* order in the *Coronaviridae*, and are divided into four types: *α, β, γ*, and *δ* (1, 2). Coronavirus subfamily members are widespread in infected birds and mammals and some respiratory and intestinal diseases (3–5). Currently, at least seven coronaviruses are known to infect humans, including respiratory syndrome coronavirus 2, human coronavirus OC43, human coronavirus NL63, human coronavirus 229E, severe acute respiratory syndrome coronavirus, human coronavirus HKU1, and Middle East respiratory syndrome coronavirus (6). Coronavirus diversity is due to the low fidelity of RNA-dependent RNA polymerase during viral coding which produces approximately 10^−3^–10^−5^ substitutions/site/year (7). Previous evidence indicated that coronaviruses undergo rapid recombination which creates new strains with altered virulence (8). Recent studies reported molecular and serological evidence of the active transmission of SARS-CoV-2-associated coronavirus (SC2R-CoV) in bats in Southeast Asia (9). Closely related coronaviruses are found in distantly related animals; the consequences of this species barrier jump may be devastating and lead to serious disease and death, e.g., SARS-CoV and MERS-CoV are zoonotic viruses that have crossed the species barrier *via* bats/palm civets and dromedary camels, respectively (10). Beta-coronavirus spill over from Hipposideridae to Rhinolophidae, and then from Rhinolophidae to civets and humans (11). Swine Acute Diarrhea Syndrome CoV is derived from the species Rhinolophus bat coronavirus HKU2 which potentially infects rodents (12, 13). Mechanistically, the species barrier jump is believed to be due to a failure in specific interactions between the viral spike protein receptor binding domain (RBD) and the host receptor, angiotensin converting enzyme 2 (ACE2) (14). This mechanism demonstrated a major tendency to jump from animals to humans.

The COVID-19 pandemic caused by SARS-CoV-2 is the latest example of a major threat to human health (15). This marks the third time a highly pathogenic coronavirus was transmitted to humans from animals (16, 17). SARS-CoV-2 is believed to have originated in bats, however the intermediate host species and the transmission mode remains unclear (18, 19). Studies confirmed that SARS-CoV-2 replicates more easily in ferrets and cats when compared with dogs, pigs, chickens, and ducks (20). The main pathophysiological feature of SARS-CoV-2 is the excessive production of inflammatory factors, leading to systemic inflammation and multiple organ dysfunction syndrome, with an acute impact on the cardiovascular system and lung fibrosis (21–24). Since SARS-CoV-2 belongs to β-coronavirus family and is not a common human pathogen, humans lack a natural immunity to SARS-CoV-2 (25). Unfortunately, the development of novel coronavirus vaccines commenced too late to effectively control the first infection wave (26). Thus far, no specific, highly effective antiviral therapies are available. The disease has rapidly spread to more than 200 countries and territories (27–30). According to World Health Organization statistics, as of February 11^th^ 2022, the total number of COVID-19 cases worldwide had reached 402,044,502, of which 5,770,023 had died (https://www.who.int/emergencies/diseases/novel-coronavirus-2019). SARS-CoV-2 is estimated to have 2–4 times more affinity for ACE2 than the SARS virus (31). Multiple mutations have been identified in the viral S1 subunit, of which three are in the RBD. This not only increases RBD affinity for ACE2 but improves viral escape from the immune system (32). Currently (September 2021), several SARS-CoV-2 delta variants have become more infectious due to mutations in the S protein RBD, and they are also ORF8-deficient. These variants have rapidly spread globally, including outbreaks in the UK (33–36), Taiwan (37), Southeast Asia (38), Germany (39), France (40), USA (41), Poland (42), and Italy (43). Strikingly, ORF8-deficient variants had spread among domestic mink and pangolin in Denmark, and were detected in humans (44, 45). Thus, ORF8-deficient variants in unknown animal reservoirs pose great challenges to human public health and safety (46), thus monitoring such SARS-CoV-2 variants is critical for the prevention and control of the COVID-19 pandemic (36).

Previous studies reported that COVID-19 severity was related to several risk factors, for example, obesity, old age, and underlying disease (47–49). A recent study suggested that when compared to the normal population, cholesterol levels were significantly lower in COVID-19 patients (50). Furthermore, high-density lipoprotein cholesterol was lower in patients with severe and critical disease than in patients with moderate or mild disease, in a study on cholesterol metabolism in mild, moderate, severe, and critical COVID-19 patients (51). Age-specific COVID-19-associated death data from 45 countries showed that the infection fatality ratio was lowest among 5–9-year-old children, with a log-linear increase by age in individuals over 30 years old (52). In Spanish subjects over 75 years old, the lethality rate approached 36% in hospitalized patients, far higher than for younger groups under the same conditions in hospitals, despite having s similar course to younger individuals (53). In addition, Richter and Sohrabi studied obese factors in COVID-19 patients when compared with the normal population. In general, they observed that obese patients were twice as likely to develop COVID-19 as those with a normal weight range (54, 55). A study of clinical characteristics on 393 patients with COVID-19 in New York City, they found that respiratory failure, a severe clinical symptom of COVID-19, was more common among the obese patient subgroup, comprising 35.8% of the patients studied in New York. In addition, they also found that after advanced age, obesity was the most common risk factor leading to severe disease and death from COVID-19 (56). In addition to these factors, male sex, diabetes, smoking, hypertension, and cardiovascular disease also affect COVID-19 risk severity.

**Figure 1:**
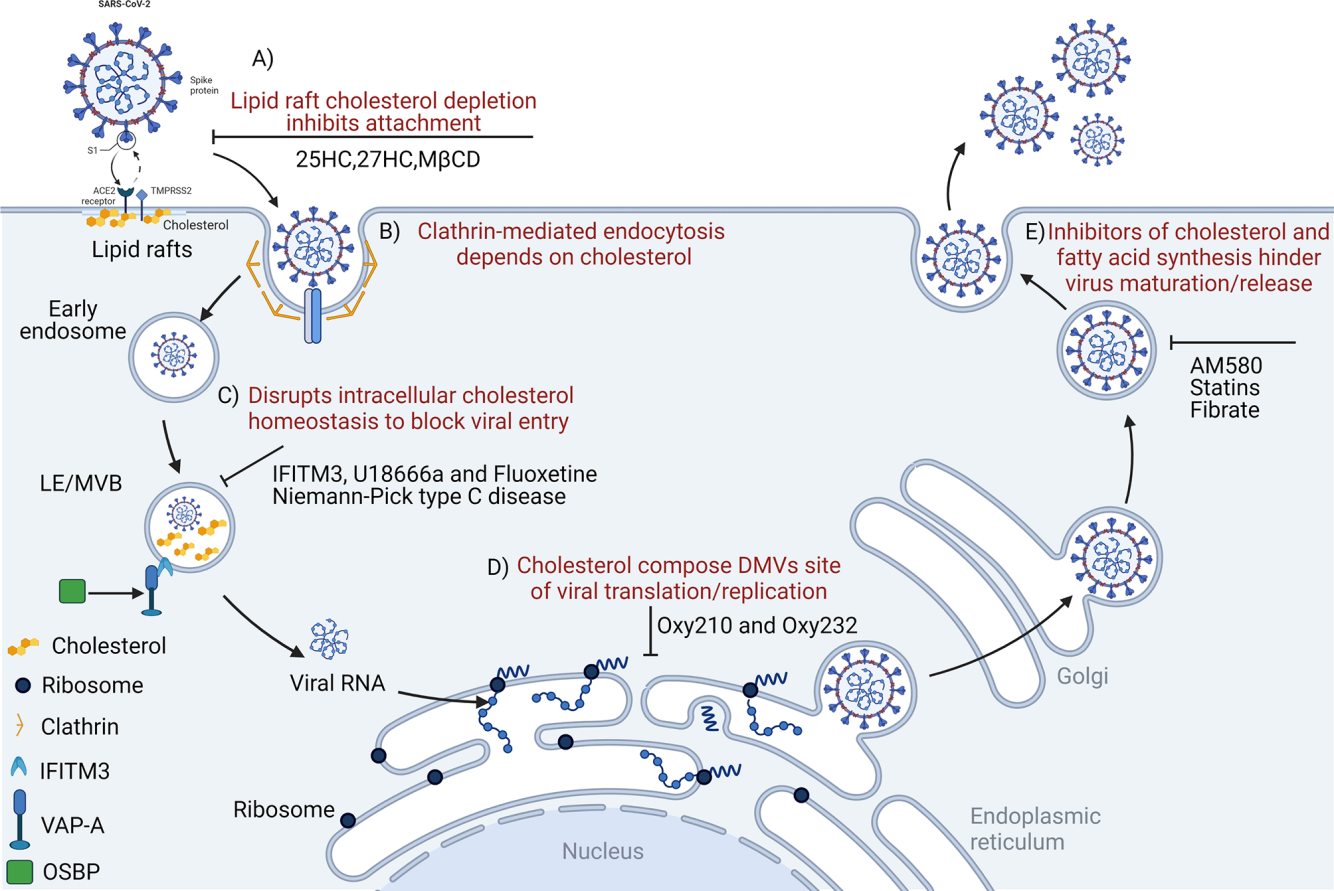
Disrupting cholesterol homeostasis interferes with critical steps during coronavirus infection. Cholesterol is important for coronavirus attachment **(A)**, endocytosis **(B)**, membrane fusion **(C)**, translation/replication **(D)**, and maturation/release **(E)**. 25HC, 25-hydroxycholesterol; MβCD, methyl-beta-cyclodextrin; LE/MVB, late Endosomes/Multivesicular Bodies; IFITM3, interferon-induced transmembrane protein-3; U18666a, an intra-cellular cholesterol transport inhibitor; DMVs, double-membrane vesicles; OSBP, oxysterol-binding protein; VAP-A, vesicle-membrane-protein-associated protein A; Oxy210, semi-synthetic oxysterols; Oxy232, semi-synthetic oxysterols; AM580, a selective retinoic acid receptor-α agonist.

Importantly, transcriptomics data indicated that host cholesterol metabolism affects virus replication (57). Cholesterol content in the plasma membrane is extremely high and is important for biochemical and biophysical functions (58). As a unique feature of mammalian membranes, host cell cholesterol is targeted by pathogens (cytosolic bacteria and viruses) for entry and egress (59–63), however, a small number of coronavirus strains are distinct in terms of their dependence on cholesterol (64). Notwithstanding, a strong relationship between cholesterol and coronavirus replication is widely documented in the literature; in some instances, cholesterol is vital for coronavirus entry, membrane fusion, translation, pathological syncytia formation and vascular pathology (65–68) **(** **Figure 1** **)**. Cholesterol metabolism may be hijacked by enveloped viruses to provide raw materials for virus particle replication, assembly, and maturation (69), e.g., Hepatitis C virus (HCV), human cytomegalovirus, and Epstein-Barr virus (70–72). For coronaviruses, cholesterol and other specific lipid requirements are required for viral replication scaffolds (73–75). Also, previous studies reported significant associations between cholesterol homeostasis and type I interferon (IFN) responses (76). Viral infections may induce host cells to alter the expression of cholesterol metabolizing enzymes and metabolites, and similarly, cholesterol metabolism can also regulate host antiviral responses (77, 78). Therefore, weaponizing host cholesterol metabolism dysregulation against coronavirus infectivity could be an effective antiviral strategy (79, 80).

Thus, cholesterol could be an important tool for the in-depth exploration of COVID-19 pathophysiology (81). The disruption of cholesterol homeostasis may interfere with critical steps of coronavirus infection, therefore understanding cholesterol functions during COVID-19 pathogenesis may generate improved prognostics and therapeutics (27). Recently, Daniloski et al. conducted a large-scale screen of > 20,000 drugs potentially used to treat COVID-19, and identified cholesterol biosynthesis pathway induction as a possible mechanism of viral inhibition (82, 83). The pharmacological inhibition of phosphatidylinositol kinases and cholesterol homeostasis reduced replication of all three coronaviruses. These findings provide important insights for an increased understanding of the coronavirus life cycle and the development of host-directed therapies (84). This review focuses on the latest scientific evidence and clarifies how coronaviruses manipulate host cholesterol metabolism to meet their own replication needs and impair host IFN responses. In addition, targeting and altering cholesterol levels in host cell membranes, and interfering with intracellular cholesterol metabolism pathways may be effective strategies in preventing early coronavirus cell entry and subsequent translation and replication. These approaches could provide foundations for the design of anti-coronavirus drug and treatment strategies (79, 85).

## The Role of Cholesterol Metabolism During the Coronavirus Life Cycle

### Molecular Mechanisms of Coronavirus Entry

Coronavirus entry into host cells is key for virus replication cycles and evading host antiviral responses (86, 87). However, coronaviruses enter cells by two ways: 1) when sufficient proteases are present on plasma membranes, viruses exploit this by fusing with the cell through the “early pathway” *via* the plasma membrane, and 2) in the absence of extracellular proteases, endosomal proteases activate the viral S protein to gain cell entry *via* the endosomal pathway (88) which is sensitive to the pH of endosome/lysosome pathways (89). Moreover, the infection efficiency of SARS-CoV in the cell in the “early pathway” is 100-1000 times higher than the endosomal pathway (90). Previous research reported that many coronaviruses, including infectious bronchitis virus (IBV), porcine hemagglutinating encephalomyelitis virus (PHEV), porcine epidemic diarrhea virus (PEDV), and feline coronavirus (FCoV) enter host cells *via* the endocytic pathway and then pass-through endosomal compartments *via* multivesicular bodies (MVBs) to enter the cytoplasm (91–94). The coronavirus S protein plays a key role in early viral infection stages and is necessary for viral entry and chemotaxis in hosts (95). This protein is a type I homotrimeric transmembrane fusion glycoprotein, composed of S1 and S2 subunits with different functions. The RBD of the S1 subunit recognizes the cell receptor, ACE2, which determines cell homogeneity and pathogenicity of coronaviruses. The S2 subunit mediates virus and host cell membrane fusion *via* a wide range of conformational rearrangements (96–98). Moreover, the S1 subunit of SARS-2-S also binds to cholesterol and possibly high-density lipoprotein (HDL) components to enhance *in vitro* viral uptake; this mechanism is mediated by the HDL scavenger receptor B type 1 (99). In addition, the SARS-CoV-2 spike protein S1/S2 boundary sequence has key roles in regulating viral entry and spread within the cell (96). More importantly, S1/S2 border sequence deletion switches SARS-CoV-2 from the plasma membrane to the endosomal fusion pathway, significantly reducing viral transmission efficiency in hamsters (100). In particular, the S1/S2 boundary sequence contains a furin protease cleavage site which pre-activates the S protein for membrane fusion, reducing SARS-CoV-2 dependence on plasma membrane proteases (e.g., TMPRSS2), thereby efficiently improving cell entry (101). When compared with other coronaviruses, murine coronavirus (MHV) is unique; its receptor binding has dual roles when gaining cell entry: the S protein N-terminal domain binds to the host receptor protein, CEACAM1a (102), then MHV uses a zinc metalloprotease for invasion and cell-cell fusion (103). This not only promotes MHV attachment to host cells, but also promotes MHV fusion with the host membrane (104). More specifically, the CEACAM1 receptor or a pH 8 induces conformational changes in the MHV S glycoprotein at 37°C. This conformational change is more conducive to triggering membrane fusion without the need to activate cleavage between S1 and S2 in advance (105).

Niemann-Pick disease type C (NPC) is a lysosomal storage disorder (106) caused by deficient lipid efflux from the late endosome/lysosome (LE/L) and induces intracellular cholesterol synthesis and transport disorders to impair viral SARS-CoV-2 infectivity *via* several lipid-dependent mechanisms (79). By intervening in the NPC1 pathway, SARS-CoV-2 is blocked from entering the host cell from the plasma membrane or endosomes/lysosomes, thus viral infectivity is weakened (107). The Ebola virus requires a functional NPC1 protein to complete its replication cycle, however, it is unclear if this is true for coronaviruses (108). Studies reported that SARS-CoV particle transport through endosomes to NPC1 positive compartments of the lysosomal system was necessary for successful infection (109). Mingo et al. showed that Ebola virus reaching NPC1-positive LE/Ls was the rate-limiting step in determining viral infection (109). Furthermore, although SARS-CoV does not require NPC1 for entry, its entry into the cytoplasm begins after colocalization with NPC1 (109). Therefore, pharmacological interventions targeting lysosomal functions could induce transient NPC1-like cells and biochemical phenotypes, which could constitute a possible rationale for COVID-19 treatment (110). Drugs such as fluoxetine not only damage LE/L acidification but also accumulate cholesterol in these compartments (111).

### Host Cholesterol in Coronavirus Entry

Coronavirus enters host cells mainly *via* plasma membrane fusion or endocytosis (112, 113). Lipid rafts participate in endocytosis-mediated processes, and function as platform and docking sites for coronavirus entry and genome release (114, 115). Cholesterol is an important component of lipid rafts; increased lipid raft formation is benefitted by increased cholesterol levels (116). Early coronavirus infection depends on lipid rafts (117) which may harbor ACE2 receptors for the SARS-CoV-2 S protein (118–121), permitting membrane rearrangements to facilitate transmissible gastroenteritis virus (TGEV) entry (122). In addition, lipid rafts act as attachment factors to promote IBV absorption before it enters the cell (119). MHV entry and membrane fusion also require lipid rafts (123). Membrane cholesterol consumption inhibits SARS-CoV-2 and other coronaviruses from fusing with cells, preventing viral entry (124, 125). By reducing plasma membrane cholesterol levels and changing lipid raft-dependent ACE2 and TMPRSS2 activities, these processes interfere with viral internalization by host cells (87, 110, 126). Therefore, cholesterol depletion from cellular membranes using e.g., methyl-β-cyclodextrin (MβCD) eliminates cholesterol in lipid rafts and significantly reduces clathrin-dependent endocytosis to significantly eliminate IBV, TGEV, and SARS-CoV infectivity **(** **Figure 1A** **)** (117, 119, 127). In addition, SARS-CoV-2 pathogenicity was significantly dependent on TMPRSS2 (128). Contributions of human ACE2 and TMPRSS2 in determining host-pathogen interaction of COVID-19 (129). The SARS-CoV-2 Omicron variant showed less efficient replication and fusion activity when compared with the Delta variant in TMPRSS2-expressed cells (130). Omicron infection was not enhanced by TMPRSS2 but was largely mediated by the endocytic pathway. The differences in pathway entry between variants may have impacted on clinical manifestation or disease severity (130). In addition, anti-androgens target TMPRSS2 and reduce SARS-CoV-2 virus entry in lung cells (131), which may at least in part explain why men with COVID-19 have a worse prognosis when compared to women (132). SARS-CoV-2 cell entry inhibition *via* TMPRSS2 was facilitated by camostat, nafamostat mesylate and alpha-1 antitrypsin (133, 134). It is therefore possible that inhibiting androgen signaling by anti-androgens could reduce TMPRSS2 expression in the lung, and concomitantly reduce viral entry. For this reason, anti-androgens are proposed as treatment options for COVID-19 (135, 136).

Infectivity is also reduced by depleting cholesterol from the viral envelope as in TGEV (127). Similarly, plasma membrane cholesterol depletion is also triggered by ACE2 displacement from lipid rafts to non-raft membrane domains, thereby reducing efficient SARS-CoV cell entry (120). Previous studies reported that 27-hydroxycholesterol (27HC) accumulation in lipid rafts caused the rapid consumption of lipid raft cholesterol, interrupted cell signal transduction in lipid raft membrane microdomains, and specifically inhibited IL-6-JAK-STAT3 signaling (137, 138). It is worth emphasizing that lipid raft destruction due to cholesterol consumption may be the main reason for inhibiting extracellular signal-regulated kinase (ERK) signaling and activation inhibition (139). Since the Raf/MEK/ERK pathway is involved in the modulation of various important cellular functions, numerous DNA and RNA viruses coopt this pathway for efficient viral propagation (140). The ERK pathway is known to be modulated during PEDV infection (141). In our previous research, we reported that IBV infection activated ERK1/2 signaling and that up-regulation of the phosphatase, DUSP6 formed a negative regulation loop (142). ERK activation is necessary for PEDV and porcine deltacoronavirus (PDCoV) replication, the suppression of viral protein expression, and viral RNA transcription *via* ERK activation inhibition (140, 143). Also, the negative regulation of the Raf/MEK/ERK signaling pathway by the MEK inhibitor, U0126 or DUSP6 upregulation significantly impairs MHV and IBV progeny production (142, 144), respectively. However, the exact mechanism whereby ERK activity regulates the replication cycle of PEDV during infection remains unclear. Therefore, the targeted regulation of lipid raft cholesterol levels may be a host defense strategy against coronavirus infection (145).

### Host Cholesterol in Viral Fusion

Along with binding to host cell receptors, viral envelope fusion with host cell membranes is critical in establishing successful coronavirus infection, especially for viral gene delivery into the cytoplasm. Coronaviruses enter cells by fusing directly with the cell surface or internalization *via* endosomal membranes (146). In general, the viral envelope contains specific cholesterol quantities; cholesterol is an important component of lipid rafts, and the fusion of viruses and host plasma membranes is affected by the ratio of membrane cholesterol to fatty acids (147). Genome-wide clustered regularly interspaced short palindromic repeats (CRISPR) screening revealed that cholesterol metabolism was a key host pathway promoting coronavirus (SARS-CoV-2, HCoV-229E, and HCoV-OC43) infections (84), whereas cholesterol dysregulation reduced viral invasion (82, 84, 100, 148). In addition, coronavirus enters cells either *via* fusion or endocytosis (149) *via* clathrin-mediated mechanisms in a cholesterol dependent manner (**Figure 1B**) (92). Cellular cholesterol homeostasis regulation, especially in endosomal compartments, exerts a significant impact on the entry stage of viral infection (108). It is because that coronavirus or coronavirus-containing MVBs *via* the endosomal cathepsin activate viral S protein to mediate the cytoplasmic release of viral nucleic acid, and artificially destroying the homeostasis of cholesterol in the endosomal membrane will inhibit this invasion step (91, 150). Therefore, the virus reprograms cholesterol metabolism to promote virus replication, or specific infection-induced host defense responses. Targeting cholesterol metabolism pathways in cells could be a potential target for interfering with “viral cargo”, and may be used as an intervention to inhibit coronavirus membrane fusion in the endosome (79). Many coronaviruses, including IBV, PHEV, PEDV, and FCoV pass through endosomal compartment *via* MVBs to enter the cytoplasm (91–94). In a previous study, IBV membrane fusion was induced in the LE/L after 1 hour post infection (91). The accumulation of cholesterol and oxidized sterols in late endosomes and MVBs also impaired virus functions, hindered viral membrane fusion, and subsequent replication (79, 151). Therefore, the destruction of cholesterol homeostasis to block viral entry exemplifies the importance of cholesterol during viral infections (150). Cholesterol function during viral invasion was extensively studied in several coronaviruses, including SARS-CoV (120), PEDV (152), MHV (123, 153), PDCoV (154), and IBV (117, 119).

Coronavirus infections may be significantly restricted by IFN-induced transmembrane proteins (IFITMs) (155). These proteins significantly inhibit endosome membrane fusion and are driven by the viral S protein (156). IFITMs inhibit viral membrane fusion before hemifusion occurs, by reducing membrane fluidity and imparting positive spontaneous curvature to outer cell membrane leaflets (157). IFITM phosphorylation status and carboxy-terminal amino acid residues are key factors determining human coronavirus entry, including, HCoV-NL63, SARS-CoV, MERS-CoV, HCoV-OC43, and MERS-CoV (158). These functional units may pass it interacts with the virus and/or host cell components of the virus entry site to regulate the fusion of the virus envelope and cell membrane (158)The vesicle membrane-associated protein A (VAPA) and oxysterol-binding protein (OSBP) jointly regulate intracellular cholesterol balance (150). IFITM3, as a member of the IFITM protein family, hinders binding of OSBP and VAPA, which not only causes abnormal cholesterol accumulation in late endosomes, but also increases membrane hardness and inhibits viral nucleic acid release **(** **Figure 1C** **)** (150).

### Cholesterol Metabolism Is Involved in Coronavirus Translation/Replication

As a positive-strand RNA virus, after internalization and un-coating, coronavirus first uses its own genomic RNA as a template to replicate and produce the polyproteins, pp1a and pp1ab *via* cap-dependent translation, and then *via* autoproteolytic cleavage, 15–16 nonstructural proteins (NSPs) (159). NSPs induce the rearrangement of cholesterol-rich lipid rafts on cell membranes, forming double-membrane vesicles (DMVs) in the cytoplasm, thereby anchoring viral replication transcription complexes (160). DMVs act as efficient replication sites for coronavirus genomic RNA and provide a safe site for viral RNA replication and translation (161). Cholesterol is also enriched in DMVs and constitutes the viral replication site of DMVs with fatty acids (80, 162). DMVs destroyed by Oxy210 (semi-synthetic oxysterol) significantly inhibit SARS-COV-2 replication *in vitro* (163) **(** **Figure 1D** **)**. Thus, disruption of lipid rafts may affect viral replication and transcriptional synthesis. Recently, it was reported that intracellular cholesterol biosynthesis and transport systems were related to virus replication (164–166). Cellular cholesterol is derived from the biosynthesis and cellular uptake of low-density lipoprotein (167–169). U18666A is a cationic amphiphilic drug affecting cholesterol biosynthesis and intracellular transport (170). Previous studies reported that cholesterol was involved in the viral life cycle of type I FCoV infection (64), and that U18666A induced cholesterol accumulation *via* NPC1 dysfunction and type I FCoV replication inhibition (144, 171, 172).

### Host Cholesterol May Not Be Involved in Coronavirus Assembly and Release

After un-coating, translation, and genome replication, virus particles assemble in the endoplasmic reticulum (ER)-Golgi intermediate compartment and are coordinated by the M protein (159, 173). For most coronaviruses, virus assembly sites contain highly active enzymes involved in the cholesterol biosynthesis pathway; these include, cholesterol-synthesizing enzyme, 3-hydroxy-3-methyl glutaryl coenzyme A reductase, and mevalonate diphospho decarboxylase (165, 174, 175). Several studies indicated that many enveloped viruses, such as human immunodeficiency virus, Dengue, Zika, and alphavirus contain cholesterol in the virion, and that viral proteins involved in virus particle assembly and budding are related to cholesterol (78, 176). Furthermore, different cholesterol levels in hosts generate different envelope cholesterol levels in alphaviruses (177). When compared with mayaro virus particles from mosquito cells, virus particle envelopes from vertebrate cells have higher cholesterol levels (178). In terms of coronaviruses, Simons et al. found that although MHV- S protein was localized to the Golgi, that contained cholesterol and lipid rafts, the assembled and released of MHV is not associated with cholesterol (167). But, cholesterol involvement in virus assembly and budding has mainly focused on viruses budding from cell membranes, however, studies on viruses budding from intracellular membranes are rare (167). Moreover, the different functional roles of cholesterol in enveloped RNA virus stability, infectivity, and assembly are not entirely clear (177, 179). Therefore, coronavirus assembly and budding may not necessarily use cholesterol on Golgi membranes, thus specific mechanisms require further study.

### Cholesterol Metabolizing Enzymes and Metabolites Combat Coronavirus Infectivity

**Figure 2:**
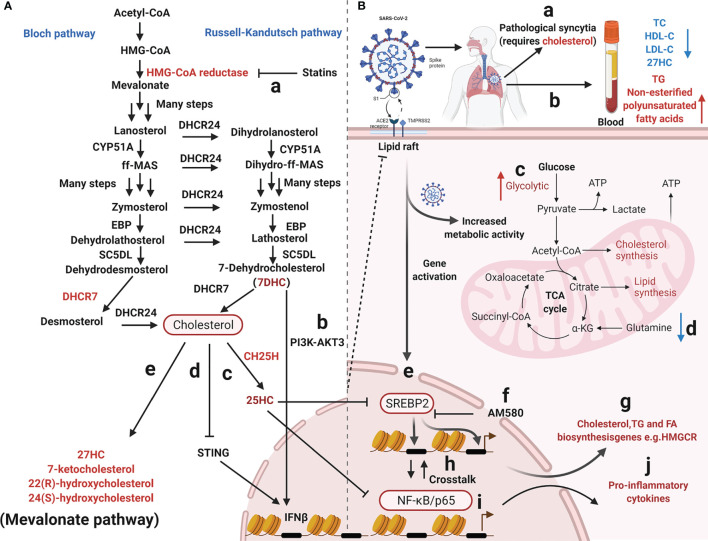
Overview of mevalonate pathway and coronavirus infection. **(A)** Cholesterol metabolizing enzymes and metabolites act against viral infectivity. Red type represents cholesterol metabolizing enzymes or corresponding natural products which may be used as drug targets or directly to exert antiviral effects. **(a)** HMG-CoA reductase regulates cholesterol biosynthesis and is targeted by statins. **(b, c, d)** The sterol metabolic network participates in interferon (IFN) antiviral responses. **(e, f)** SARS-CoV-2 propagation in cultured cells is inhibited by various cholesterol molecules and semi-synthetic oxysterols. The detailed steps of the cholesterol synthesis pathway can be found in (180–182). **(B)** Cholesterol metabolism reprogramming and antiviral responses after viral infection. **(a)** Cholesterol promotes pathological syncytial formation during SARS-COV-2 infection. **(b)** Serum TC, TG, and non-esterified polyunsaturated fatty acid levels are remodeled in COVID-19 patients. **(c, d)** SARS-CoV-2 infection increases glucose entry into the TCA cycle *via* increased pyruvate carboxylase expression and reduced oxidative glutamine metabolism, while maintaining reductive carboxylation. **(f)** SREBP-dependent lipidomic reprogramming is a broad-spectrum antiviral target, AM580 strongly inhibits coronavirus replication by interacting with SREBP-2. **(e, h, g, i, j)** COVID-19-activated SREBP-2 disturbs cholesterol biosynthesis, leading to a cytokine storm. Importantly, SREBP-2 activity is regulated by crosstalk between cholesterol consumption and NF-κB expression *via* several inflammatory response processes induced by SARS-CoV-2 infection. Red arrows represent upregulation and blue arrows represent downregulation. Acetyl-CoA, Acetyl-Coenzyme A; HMG-CoA, 3-hydroxy-3-methylglutaryl-coenzyme A; EBP, Δ(7)-isomerase; DHCR24, 3-β-hydroxysteroid-Δ-24-reductase; SC5DL, Sterol C5-desaturase; DHCR7, 7-dehydrocholesterol reductase; CH25H, cholesterol-25-hydroxylase; 25HC, 25-hydroxycholesterol; 27HC, 27-hydroxycholesterol; IFNβ, Interferon-β; TC, Total cholesterol; HDL-C, High-density lipoprotein cholesterol; LDL-C, low-density lipoprotein cholesterol; TG, Triglyceride; ATP, Adenosine triphosphate; SREBP-2, Sterol regulatory element-binding protein 2; AM580, a selective RARα agonist; HMGCR, 3-Hydroxy-3-Methylglutaryl Coenzyme A Reductase; NF-κB, Nuclear transcription factor-κB.

Coronavirus infection induces host cells to alter the expression of certain cholesterol metabolizing enzymes and metabolites which may exert antiviral effects **(** **Figure 2A** **)** (77). Indeed, both 25-hydroxycholesterol (25HC) and 27-hydroxycholesterol (27HC) are physiologically produced by the enzymatic oxidation of cholesterol and may be used to inhibit enveloped and non-enveloped human viruses (183, 184) and highly pathogenic viruses, including Zika (185), mammalian reovirus (186), Lassa virus (187), encephalomyocarditis virus (188), porcine reproductive and respiratory syndrome virus (189). A recent study reported that the 25HC treatment of mice infected with SARS-CoV-2 significantly reduced virus numbers in the lungs and trachea (148). On the one hand, cholesterol is transformed into 25HC by Cholesterol 25-Hydroxylase (CH25H). By obstructing membrane fusion, 25HC exhibits extensive anti-coronavirus activity (125, 184). Similarly, the internalization of 25HC aggregates in late endosomes may inhibit spike protein-catalyzed membrane fusion of SARS‐CoV‐2 by blocking cholesterol export (124); however, CH25H consumes available cholesterol on the plasma membrane to suppress virus-cell fusion (125). These data indicate that membrane-modifying oxysterols are possible antiviral therapeutics, thereby inhibiting SARS-CoV-2 and other coronaviruses **(** **Figure 1A** **)** (153). However, it is possible to obstruct PDCoV proliferation using CH25H which acts as a host restriction factor, but this inhibition is not entirely dependent on its enzymatic activity (190). The junction adhesion molecule-A and the cation independent isoform of the mannose-6-phosphate receptor are two key replication molecules common to all viruses that use adhesion molecules and the endosomal pathway to enter and diffuse target cells. Both molecules are downregulated by 25HC and 27HC (191). Previous studies suggested that SARS-CoV-2 propagation in cultured cells was inhibited by various cholesterol molecules, including natural oxysterols, 7-ketocholesterol, 22(R)-hydroxycholesterol, 24(S)-hydroxycholesterol, and 27HC **(** **Figures 2A-e, c** **)** (163). At effective concentrations, 25HC, 7-dehydrocholesterol (7DHC), and 27HC were non-toxic natural products, with potentially curative applications for emerging virus infections, such as SARS-CoV-2 (192), human immunodeficiency virus, Ebola virus, Nipah virus, Rift Valley fever virus, and Zika (153).

### The Sterol Metabolic Network Participates in Host-Immune Responses

All coronaviruses have a similar infection mechanism, which successfully manipulates host cell functions. One strategy to suppress the host innate immune response to evade antiviral responses, is shielding RNA intermediates in replication organelles (193, 194). In fact, coronaviruses with +RNA genomes, which duplicate solely in the cytoplasmic matrix, and modify the inner membranes of cells to form virus duplication bases, also known as “replication factories” or “replication organelles”. While varying in morphology and membrane composition, these structures appear to centralize viral replication machinery, intermediates, and products in membrane-bound vesicles or invaginations, and are beyond the reach of innate immune sensors in the cytosol. Thus, viral infection outcomes are determined by metabolic interactions between hosts and viruses (195). Cholesterol is a crucial component of cell membranes and lipid rafts. Cholesterol metabolism contributes to the formation of immune synapses and downstream signal transmission (196).The host’s defenses against virus infection requires IFN-mediated cholesterol biosynthesis and the formation of immune synapses, and also host innate immune metabolic regulators as potential antiviral strategies (197).

Two molecules, sterol regulatory element-binding protein-2 (SREBP-2) and 3-hydroxy-3-methyglutaryl CoA reductase (HMGCR), have significant roles in the cholesterol biosynthetic pathway; SREBP-2 is the master transcriptional regulator of cholesterol biosynthesis (**Figures 2B-e, g**) and HMGCR is a rate-limiting enzyme for cholesterol synthesis (198). SREBP-2 cleavage and HMGCR degradation are two major feedback regulatory mechanisms governing cholesterol biosynthesis **(** **Figures 2A-a** **)** (58). Recently, the sterol metabolic network was shown to participate in interferon (IFN) antiviral responses (76, 199). Studies have reported that the IFN regulatory loop mechanism downregulates sterol biosynthesis, linking innate immune responses to viral infection, *via* sterol metabolism regulation (171). After viral infection, the infected cells produce high IFN levels, thereby reducing enzyme expression in the cholesterol pathway (171). On the one hand, the sterol metabolic network is involved in IFN antiviral responses (76) with reduced flux through the mevalonate pathway leading to upregulation of type I IFN responses (172) **(** **Figures 2A-b, d** **)**. IFN-γ induces proteasomal degradation of HMG-CoA reductase and the rapid proteasomal elimination of HMG-CoA reductase by IFN-γ in primary macrophages which requires endogenous 25HC synthesis (200). On the other hand, cholesterol metabolism and mevalonate pathways are crucial for regulator T-cells which efficiently drive regulatory T cell proliferation and enhance and stabilize their suppressive capacity (201, 202). In particular, LKB1 triggered activation of the mevalonate pathway by upregulating IFN-γ and IL-17A levels, which were essential for the stabilization of T regulatory cells (201). Cholesterol is required for SARS-CoV-2 to form pathological syncytia which is believed to help replicate and evade host immune responses (67). For example, cholesterol biosynthesis pathways are affected by SARS-CoV which regulate levels of SREBP2, S1 protein, peroxisome proliferators-activated receptors γ (PPARγ), diacylglycerol acyltransferase-1or cholesterol efflux regulatory protein (203–205). COVID-19-activated SREBP2 disturbs cholesterol biosynthesis (**Figures 2B-e**), leading to a cytokine storm (50) **(** **Figures 2 B-j** **)**. Importantly, SREBP-2 activity is regulated by crosstalk between cholesterol consumption and nuclear factor κ-B (NF-κB) expression from various inflammatory response processes induced by SARS-CoV-2 infection (**Figures 2B-h**) (50). In addition, a metabolic configuration is induced by SREBPs where glucose is metabolized *via* the citrate malate shuttle, thus enabling natural killer cell growth, proliferation, and function (206). Therefore, SREBP-dependent lipidomic reprogramming may be viewed as a broad-spectrum antiviral target (203). 25HC exerts inflammatory properties and significantly attenuates proteolytic processing of SREBP2 **(** **Figures 2A-c** **)**, thereby inhibiting the isoprenoid branch of the mevalonate pathway (207). 25HC also amplifies inflammatory signals (207), with growing evidence suggesting it has a broad impact on innate and adaptive immunity (195, 208–213), including antiviral immunity, inflammasome activation, and antibody class switching (214), In addition, lung-selective 25HC nano-therapeutics may function as inhibitors of COVID-19-mediated cytokine storms (215). IFN-β production may be regulated by targeting the 7-dehydrocholesterol reductase (DHCR7) and adding 7-dehydrocholesterol (7-DHC), an intermediate natural product in the cholesterol metabolism pathway (77) **(** **Figures 2A-b** **)**. Moreover, it is possible to enhance anti-viral immunity by promoting serine/threonine kinase 3 (AKT3) activation (77); a positive feedback loop is formed *via* type I IFN signaling and 7DHC accumulation to amplify innate immune responses and control viral infection by activating AKT3. Also, the emergence of highly pathogenic viruses may be inhibited by DHCR7 inhibitors and 7-DHC (216). York et al. suggested that a reduction in cholesterol biosynthesis was a key event in inducing antiviral responses in virus-infected cells (172) and that decreased cholesterol biosynthesis facilitated anti-viral signaling by the stimulator of interferon gene (STING) in the ER **(** **Figures 2A-d** **)** (216). Although cholesterol is a crucial component of immune cell membranes, cholesterol accumulation in lymphoid organs promoted T cell priming and stimulated the production of the B cell growth factors, Baff and April (217). Ito et al. (2016) reported that defects in cholesterol metabolism in CD11c^+^ immune cells resulted in impaired antigen presentation and ultimately autoimmune disease (218). Excessive cholesterol may exert immune dysfunction and promote excessive pulmonary and systemic inflammatory responses (219).

### Cholesterol-Modifying Drugs Inhibit Coronavirus Replication

Recently, several commonly prescribed medications were shown to interfere with sterol biosynthesis, including haloperidol, aripiprazole, cariprazine, fluoxetine, trazodone, and amiodarone (220). Cholesterol-modifying drugs exert anti-viral roles by reducing the absorption or synthesis of systemic cholesterol or directly changing cholesterol levels in target cell membranes (219) **(** **Figure 1A** **)**. It is possible to alter the SARS-CoV-2 cycle *in vitro* and *in vivo* using various cholesterol-modifying drugs (e.g., AM580 is a selective retinoic acid receptor alpha (RAR-α) agonist, fibrates, and statins) which hinder fatty acid and cholesterol synthesis **(** **Figure 1E** **)** (221). In fact, cholesterol-binding agents, including statins or MβCD, affect cholesterol and destroy lipid rafts, thereby damaging coronavirus adhesion and binding properties (119, 222). Moreover, these compounds also block key downstream virus infectivity molecules, reduce proinflammatory tumor necrosis factor-α (TNF-α) and IL-6 levels, and/or affect autophagic processes in viral replication and clearance (222). It is worth noting that cholesterol, fatty acids, cytosolic phospholipase A2α (cPLA2α), and fatty acid synthase contribute to SARS-CoV-2 DMV formation (204, 223). For example, fenofibrate (reduces triglyceride and low-density lipoprotein cholesterol levels inhibit SARS-CoV-2 replication and pathogenesis by affecting lipid metabolism pathways in the lung cells of patients with COVID-19 (224). AM580 is a retinoid derivative which interacts with N-terminal SREBP to block lipogenic transactivation (203). Statins reduce intra- and extra-cellular cholesterol by targeting HMGCR **(** **Figures 2A-a** **)** (225, 226), thereby affecting viral infection, immunity, and inflammation (219). Statins may also limit inflammation by altering HMGCR mediators in the cholesterol biosynthesis pathway (227, 228). These anti-inflammatory properties are considered statin’s core protective effects in cardiovascular disease, in addition to lowering cholesterol levels (225). Wang et al. suggested that high cholesterol levels increased entry of pseudotyped SARS-CoV-2 and the infection of virus particles, and more of the receptor ACE2 can be recruited to the internalization site (229). Statins ability to decrease lipids, enhance protective immune responses, and exert anti-inflammatory properties are beneficial during SARS-CoV-2 infections (219). Statin therapy was previously reported to increase blood clearance rates in chronic HCV infections and reduce mortality and intubation requirements during influenza infection (118). Statins, especially pitavastatin, may significantly inhibit activity of SARS-CoV-2’s main protease, Mpro, which has a greater binding energy than proteases or polymerase inhibitors (230). Decreasing cellular cholesterol may also trigger the intake of more cholesterol from the blood, reducing serum HDL-cholesterol (HDL-C) and LDL-C levels. As cholesterol-lowering drugs, statins are widely used in cardiovascular and metabolic diseases (231, 232). They inhibit inflammation by reducing cholesterol and phospholipid deposition in blood vessels. Thus, anti-inflammatory molecules provide protective effects in cardiovascular diseases, and do not just lower cholesterol (225).

### Cholesterol as a Potential Marker for Monitoring COVID-19

SARS-CoV-2 infection reshapes cholesterol metabolism *via* gene activation and increased host metabolism activity (23) **(** **Figure 2B** **)**. Briefly, SARS-CoV-2 infection disturbs cholesterol biosynthesis by activating SREBP-2 and affecting glucose or glutamine metabolism (50, 233) **(** **Figures 2B-e, c, d** **).** Clinical data has also indicated that lipid disorders may facilitate increased COVID-19 mediated pathogenicity, therefore lowering cholesterol levels may inhibit SARS-CoV-2 replication and viral loads in patients (51, 65). When compared with healthy individuals, patients with dyslipidemia-related diseases are more likely to be infected by SARS-CoV-2 (234, 235). Also, SARS-CoV-2 infection caused some COVID-19 patients to have lower serum cholesterol levels (e.g., 27HC, total cholesterol, high density lipoprotein cholesterol, and low-density lipoprotein cholesterol), while triglyceride and non-esterified polyunsaturated fatty acid levels were up-regulated **(** **Figures 2B-b** **)** (27, 236–238). In particular, decreased serum HDL-C levels are positively correlated with COVID-19 infection severity (239). As infection worsens, serum TC and HDL are lowered, but upon recovery, cholesterol levels return to normal (65, 239–243). This may be due to SARS-CoV-2 S proteins affecting HDL functions by removing lipids from HDL and remodeling its composition/structure (243), potentially affecting virus clearance in infected patients (244). Thus, serum cholesterol and lipoprotein marker monitoring may have an important clinical value for COVID-19 risk prediction (115). Increased triglyceride/HDL-C ratios may be useful for the early identification of patients with high risk and poor outcomes (245, 246). Moreover, in patients with severe disease, significantly elevated serum HDL levels are associated with favorable outcomes (112). HDL-C levels decrease significantly in critically ill COVID-19 patients and are negatively correlated with C-reactive protein and IL-6 levels, however lymphocyte levels are increased with increased HDL-C levels, which positively correlate with the COVID-19 severity (247). Therefore, LDL-C levels may be used as predictors of COVID-19 progression and risk assessment (248).

SREBP is a membrane junction protein attached to the ER and nuclear envelope (249); it regulates the effective synthesis of fat and cholesterol and plays important roles in maintaining energy homeostasis **(** **Figures 2B-g** **)** (250, 251). The SREBP protein family regulate lipid cholesterol and fatty acid gene expression *via* mitogen-activated protein kinase (MAPK) signaling (252). A recent study reported that SREBP-2 C-terminal fragment was detected for the first time in the blood of patients with COVID-19. Based on data from clinical samples, SREBP-2 C-term was suggested as a reference indicator to assess disease severity after SARS-CoV-2 infection (50). SREBP-2-dependent lipidomic reprogramming is a broad-spectrum antiviral target, with SREBP-2 activation correlating with COVID-19-induced cytokine storm activation (235). AM580 strongly inhibits coronavirus replication by interacting with SREBP-2 (203, 204).

## Conclusions

Viruses are intracellular parasitic pathogens. They exploit host nutrients and metabolites to accommodate their survival and are highly adaptable molecules in escaping host antiviral responses. Therefore, interventions in host specific metabolic pathways could become potential antiviral targets (80, 203, 205, 253). Potential cholesterol-modifying drugs exert broad-spectrum antiviral effects by inhibiting activities of key rate-limiting enzymes in the mevalonate pathway, and also SREBP proteins which regulate host cholesterol homeostasis, thereby affecting coronavirus entry, membrane fusion, and pathological syncytia formation (204). Thus, cholesterol metabolism disorder is a double-edged sword; it affects the normal physiological functions of cells, however, weaponizing cholesterol dysregulation in local cell environments such as lipid rafts or endosomes could inhibit coronavirus replication. Therefore, the development of selective cholesterol-modifying drugs targeting key cellular components such as lipid rafts and endosomes in infected cells could be a promising antiviral strategy for the early stages of coronavirus infection.

## Author Contributions

CD and XQ conceived the review concept and drafted the article. JD and HW wrote the original draft and prepared figures. YL, LT, YS, CS, and WL edited and reviewed the manuscript. All authors read and agreed to the final published version of the manuscript.

## Conflict of Interest

The authors declare that the research was conducted in the absence of any commercial or financial relationships that could be construed as a potential conflict of interest.

## Publisher’s Note

All claims expressed in this article are solely those of the authors and do not necessarily represent those of their affiliated organizations, or those of the publisher, the editors and the reviewers. Any product that may be evaluated in this article, or claim that may be made by its manufacturer, is not guaranteed or endorsed by the publisher.
